# Skin graft associated with platelet-rich plasma in correcting extensive injuries resulting from the resection of skin cancer chemically induced in rats

**DOI:** 10.1590/ACB361203

**Published:** 2022-01-05

**Authors:** Josiane Morais Pazzini, Stella Habib Moreira, Pedro Cassino, Marjury Maronezi, Michelle Zangirolami, Jorge Luis Alvaréz Gomés, Paulo Henrique Bertolo, Michelle do Carmo Pereira Rocha, Sonia Prince Fiebi, Caroline Kajiura, Filippo Janoni Bernardes, Caio Bustamante, Andrigo Barboza De Nardi

**Affiliations:** 1 DVM, PhD. União das Faculdades dos Grandes Lagos - Sao Jose do Rio Preto (SP), Brazil.; 2 Fellow PhD degree. Postgraduate Program in Veterinary Surgery - Department of Clinical and Veterinary Surgery - Faculdade de Ciências Agrarias e Veterinárias – Universidade Estadual Paulista - Jaboticabal (SP), Brazil.; 3 Fellow PhD degree. Postgraduate Program in Veterinary Surgery - Department of Clinical and Veterinary Surgery - Faculdade de Ciências Agrarias e Veterinárias – Universidade Estadual Paulista - Jaboticabal (SP), Brazil.; 4 Fellow PhD degree. Postgraduate Program in Veterinary Surgery - Department of Clinical and Veterinary Surgery - Faculdade de Ciências Agrarias e Veterinárias – Universidade Estadual Paulista - Jaboticabal (SP), Brazil.; 5 Graduate student. Faculdade de Ciências Agrarias e Veterinárias – Universidade Estadual Paulista - Jaboticabal (SP), Brazil.; 6 Full Professor. Department of Clinical and Veterinary Surgery - Faculdade de Ciências Agrarias e Veterinárias – Universidade Estadual Paulista - Jaboticabal (SP), Brazil.

**Keywords:** Angiogenesis Inducing Agents, Granulation Tissue, Platelet-Rich Plasma, Skin Transplantation, Surgical Flaps, Rats

## Abstract

**Purpose:**

To evaluate whether using platelet-rich plasma (PRP) in the graft recipient bed after the resection of a neoplasia can influence its recurrence because this product stimulates angiogenesis, mitogenesis and chemotaxis.

**Methods:**

A study with 30 rats Wistar (*Rattus norvegicus albinus*), which were separated into group A (induction of carcinogenesis, PRP in the postoperative period) and group B (induction of carcinogenesis, absence of PRP in the postoperative period), with 15 animals in each. Carcinogenesis was induced on the skin of the animals’ chest by the topical application of 0.5% dimethylbenzantracene (DMBA) diluted in acetone. After surgical resection of the induced neoplasia, PRP was used to stimulate angiogenesis before surgical wound synthesis. Data on the control and experimental groups and macroscopic and microscopic variables were evaluated using analysis of variance and the Tukey’s test (5%).

**Results:**

It was possible to determine that the use of PRP is good in reconstructive surgeries, but it is contraindicated in patients during tumor resection, as it can cause changes in the surgical bed, in addition to stimulating recurrences and metastases.

**Conclusions:**

PRP may interact with tumour cells that were in the recipient site of the surgical wound during the resection of a neoplasia, and a local recurrence process can be triggered by applying this product.

## Introduction

The growth of reconstructive surgery in veterinary medicine has occurred because of the increased survival of patients and owners seek the treatment and diagnosis of their animals’ diseases. It is important to emphazise that reconstructive surgery aims to repair defects that have a great loss of continuity of the integument and compromise healing, and to provide the patient with better aesthetic and functional results^1^^,^ 2.

According to Almeida *et al*.^3^, vascularization of the flaps and grafts is important and much discussed, besides one cause of complications in reconstructive surgeries. The grafts are deprived of arteries and veins in their constitution. Therefore, they are impaired by necrosis after being implanted in a recipient bed^4^.

In addition, the use of high-dose platelet-rich plasma (PRP) in surgical wounds has been reported in several studies, which include the use of substances that improve wound healing by stimulating angiogenesis^5^^,^ 6. Many studies report the effectiveness of PRP in healing when applied in reconstructive surgeries^5^^-^ 10. Therefore, the efficacy of the use of PRP is proven through numerous studies. However, whether this platelet-derived product could be used after resection of tumours has not been studied.

Vascular endothelial growth factors and epithelial growth factors from platelets stimulate angiogenesis^11^^,^ 12. It has not been scientifically proven whether these factors interact with tumour cells that may remain in the recipient site of a surgical wound during the resection of a neoplasia, and thus could trigger a local recurrence with the application of the product.

The most reported complications in reconstruction surgical procedures are related to necrosis, which occurs due to the absence of neovascularization^13^^,^ 14. However, studies that prove its efficacy in healing were conducted in healthy experimental models that mimicked extensive wounds from trauma and congenital anomalies^6^^,^ 15 ^,^ 16. Therefore, the purpose of this study was to evaluate whether using PRP at the graft receptor site after the resection of a chemically induced neoplasia may influence a relapse, because of the properties of PRP in stimulating angiogenesis, mitogenesis and chemotaxis.

## Methods

The surgical procedures of this study were conducted at Hospital Veterinário “Governador Laudo Natel”, Universidade Estadual Paulista “Júlio de Mesquita Filho” (UNESP), School of Agricultural and Veterinarian Sciences, after evaluation and approval by the Ethics Committee on the Use of Animals (CEUA), with protocol number: 008102/17, in accordance with the rules issued by the National Council for the Control of Animal Experimentation (CONCEA). The slides for histological analysis were prepared at the Laboratory of Veterinary Immunohistochemistry, at the same institution.

### Experimental group

The study was conducted on 30 healthy male rats (*Rattus norvegicus albinus*, Wistar) with mean age of 21 days and weighing approximately 30 g. They were obtained from Central Biotherm of the Faculty of Veterinary Medicine (UNESP–Campus Botucatu; Botucatu, Brazil). The groups contained 15 animals each and comprised group A (induction of carcinogenesis, use of PRP postoperatively) and group B (induction of carcinogenesis, absence of PRP in the postoperative period).

### Induction of carcinogenesis and the monitoring of the animals

The inducer of 0.5% of 7,12-dimethylbenzanthracene (DMBA) was administered in a single dose (0.05 mL) by the intradermal route, which was adapted from the method described by Mainenti and Rosa^17^, who used a cervical incision and implanted DMBA pellets in the salivary glands of rats. This method was also described by Ebling *et al*.^18^ and by Hindy *et al*.^19^, who reported the inoculation of DMBA powder by using a modified needle. For the safety of the team, the procedure was conducted in a laminar flow chamber and with individual protection, because the DMBA is a highly carcinogenic substance. The size of the lesion induced by applying DMBA should be approximately 0.5 mm^20^.

**Figure 1:**
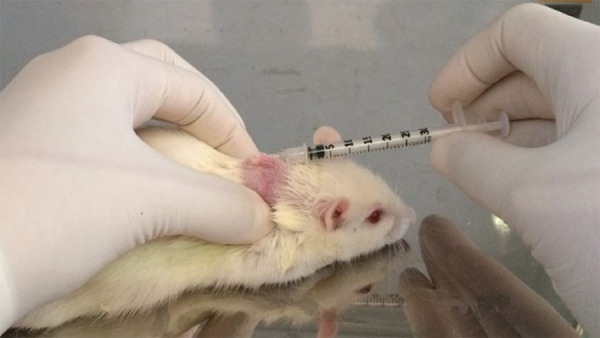
Carcinogenesis induction to determine the malignancy of the induced lesion.

Throughout the experimental period, the animals were observed daily to evaluate macroscopic changes due to the process of carcinogenesis induction. In addition, elastography was conducted at the site of carcinogenesis induction to determine the malignancy of the induced lesion (Fig. 1). After the period of induction of carcinogenesis and delimitation of the tumour lesion, the animals were submitted to the surgical procedure for tumour resection and correction of the surgical wound using a cutaneous mesh graft and using PRP.

### Surgical procedure

The protocol for obtaining autologous PRP was conducted as described by literature^21^. For the surgical procedure, a wide trichotomy of the dorsal aspect of the thorax was formed. Antisepsis was then administered with chlorhexidine and 90% alcohol solution in the donor bed region and the recipient bed.

Anaesthesia induction and maintenance were accomplished with 1–3% isoflurane diluted in 100% oxygen using an anaesthetic vaporizer to produce the surgical anaesthetic plane^22^.

A surgical pen and a sterile ruler were used to demarcate the neoplastic lesion in the thoracic region in the dorsal portion. With the aid of a scalpel blade (number 15), the neoplastic lesion was excised with a 0.5-cm margin of safety in the shape of a square. A cutaneous graft was subsequently obtained in the dorsal region of the thorax. A cutaneous incision measuring 1 cm^2^ was formed, and measured 6 cm in the caudal direction to the spinal process of the first cervical vertebra. The depth limits were determined based on the anatomical references described in the literature^13^. It was important that the size of the graft was equal to the defect created in the receiving reader. After incising the skin, the graft was carefully dissected and removed from the donor bed. This step was finished by removing fat and subcutaneous residues from the fragment and making slits in the graft. Sutures (4-0 nylon) in a single standard pattern were first distributed at the vertices of the square between the tissue of the donor bed and the recipient, and always applied in the graft direction to the skin of the recipient bed to avoid graft movement at the moment of the suture. In sequence, the remainder of the surgical wound was synthesized. Compressed gauze was applied with the gauze sutured to the surgical wound bed.

The donor bed wound in the right thorax was submitted to dermography in a geometric figure closed pattern with 4-0 nylon. The sutures began at the vertices of the defect and converged towards the center of the surgical wound. The end of the surgical scar had two inverted triangles.

The autologous PRP gel was applied with the aid of the scalpel handle in group A, before the synthesis of the lesion. It was homogeneously distributed between the graft and subcutaneous of the recipient bed. The animals of group B underwent synthesis of the graft in the recipient bed without the application of any product. All animals received meperidine at a dose of 20 mg/kg subcutaneously, every 12 hours, for seven days.

In all animals of both groups, dressings were applied in the immediate postoperative period, changed on the third, seventh and 15th days, and evaluated by blind observer with regard to the cicatrization aspect (e.g., exudate, colour, oedema)^23^. At the end of this period, the surgical site was macroscopically evaluated to monitor the development of a possible recurrence through the use of PRP.

### Macroscopic evaluation of tumour recurrence

The animals were evaluated for 24 weeks; within this time, a local recurrence and metastasis could occur^24^. Chest X-ray and abdomen ultrasound imaging were used to evaluate metastasis. After this period, the animals were euthanized for microscopic evaluations of the site in which the graft had formed.

The macroscopic evaluation of relapse consisted of assessing skin integrity and the presence of nodulations and evaluating the recurrence interval after the surgical procedure and the percentage of animals that presented recurrence^25^. The integrity of the skin was graded by aspect (normal and intact aspect = 0; scaly = 1; or nodular = 2); by nodulation (absent = 0; or present = 1); and by lesion size, which was measured with the aid of a calliper.

### Euthanasia and the microscopic evaluation of tumour recurrence

At the end of postoperative week 24, the animals were euthanized to harvest material for the microscopic evaluation. Euthanasia was done according the CONCEA.

The wounds were excised with a 1-cm margin of whole skin around the lesion. Each fragment was individually identified, fixed in paperboard, and placed in 10% formalin solution. After 48 h, the solution was replaced with 70% alcohol. Samples were processed, based on the conventional routine histological processing, inclusion in paraffin blocks, and histological sections. These samples were cut 2-μm thick in a microtome, and then incubated overnight at 37°C, after hydration with increasing dilutions of alcohol and diaphanization in xylol, and obtaining specific reactions.

The sections were stained with hematoxylin and eosin staining (H&E) for the evaluation of the gradation of epithelial atypia and healing of the lesion. The presence of mononucleates, polymorphonucleated cells, proliferation of fibroblasts, haemorrhage and necrosis was evaluated^26^. In addition, differentiation of the epithelium was evaluated by using an immunohistochemical method^27^, and CD31 proliferation^28^ was evaluated by using the monoclonal antibody Ae1/Ae3.

A pathologist evaluated the gradation of epithelial atypia of the neoplasm and the surgical scar bed. This evaluation followed the criteria proposed by Bánozci *et al*.^29^: cell pleomorphism (absent = 0; discrete = 1; moderate = 2; intense = 3); parakerotic hyperkeratosis (absent = 0; discrete = 1; moderate = 2; intense = 3); and spongiosa (absent = 0; discrete = 1; moderate = 2; intense = 3). Based on the number of epithelial atypia criteria, the gradation was classified as mild (i.e., up to two criteria), moderate (i.e., three or four criteria), or intense (i.e., five or more criteria).

For the immunohistochemical study, the cuts were spread on previously cleaned and degreased glass slides with commercial adhesives (3-aminopropyltriethoxysilane [Sigma Chemical, St. Louis, MO, United States of America]). The obtained cuts were sent for the antibodies process specific for several antigens (Table 1). The detection system used was based on the manufacturer’s instructions.

**Table 1:** Antibodies used in the immunohistochemical reaction in rat.

Antibody	Clone	Manufacturer	Dilution	Antigenic recovery	Incubation	Detection system
Ae1/Ae3	Monoclonal, Ae1/Ae3	ImPathA	1:400	Novocastra epitope retrieval solutions (pH=9; waterbath; Leica Biosystems New Castle Ltd., United Kingdom)	45 min	Novolink Polymer Detection Systems (Leica Biosystems, United Kingdom)
CD31	Monoclonal, JC70A	Dako	1:50	Pepsina porcine gastric mucosa (Sigma Life Science, United Kingdom)	45 min	Novolink Polymer Detection Systems (Leica Biosystems, United Kingdom)

The data obtained using the Ae1/Ae3 antibody were evaluated by means of photomicrographs obtained by the optical microscope at x4 magnification. The images were subseqeuently analysed using ImageJ software with the plug-in Threshold Color to obtain the percentage of the total area of cell differentiation through the analysis of automated particles, based on the selection and the measurement of the areas by color^27^.

The angiogenic index for CD31 was determined by the microvascular counting technique, as recommended by the literature^28^^,^ 29. The vessels were counted in five fields, which were previously selected, with high-vascular density at x400 magnification using an optical light microscope. A reticulum was adapted for stereology with the aim of avoiding the retelling of structures. The microvascular count was determined twice by a single evaluator at two different times. It is expressed as the mean number of vessels in each case studied.

### Statistical analysis

Statistical analysis of the control and experimental groups regarding macroscopic variables and microscopy data were submitted to statistical analysis by the analysis of variance (ANOVA) and Tukey’s test (5%). In the microscopic evaluation, the correlation between the number of vessels and the degree of epithelial atypia were subsequently conducted using Pearson’s correlation. Cell differentiation data were compared by an analysis of variance (F-test) for a completely randomized design with two groups and 10 replicates per group, considering p-value equal to or less than 0.05. The comparison between groups A and B in relation to categorical variables was evaluated by the Kruskal-Wallis test, with the subsequent use of the Dunn’s multiple comparison test. Data from the immunohistochemical evaluation of Ae1/Ae3 were compared by ANOVA (F-test) for a completely randomized design with two groups and 10 replicates per group, based on p ≤ 0.05. Data from the immunohistochemical evaluation using CD31 also underwent ANOVA with two groups, mean of five replicates per group, and significance level of 5%. For these analyses, the general linear models procedure in Statistical Analysis System (SAS) software (SAS 9.1, SAS Institute, Cary, NC, United States of America) was used.

## Results

**Figure 2:**
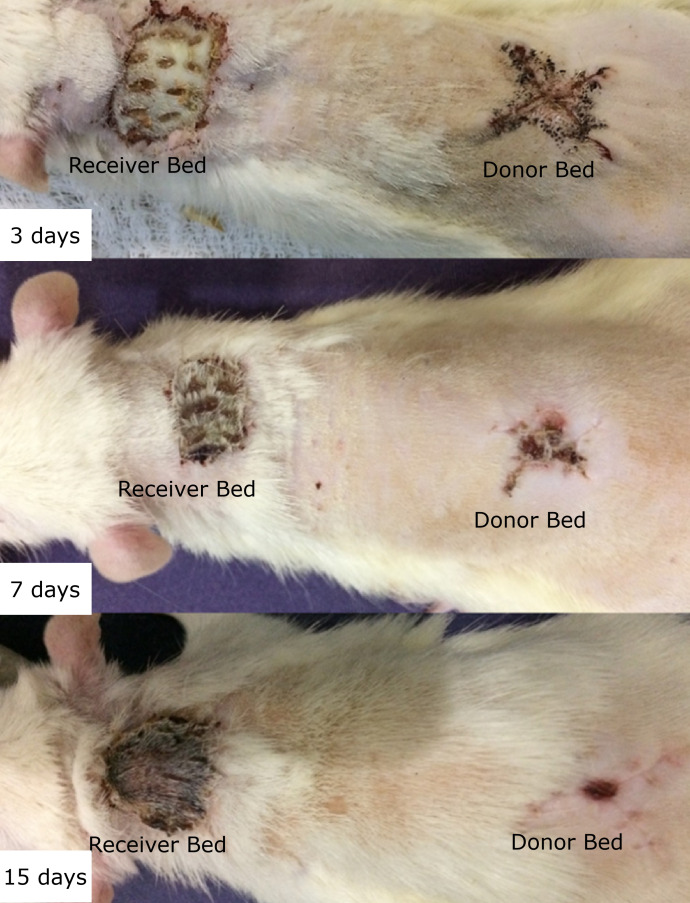
The results of the macroscopic variables (i.e., exudate, oedema, coloration, and cosmetic appearance) used to assess healing on postoperative-days 3, 7 and 15 were insignificant between the groups.

The results obtained in this study are a reference to the application of PRP in reconstructive surgery. The macroscopic variables (i.e., exudate, oedema, coloration, and cosmetic appearance) used to assess healing on postoperative-day 3, 7 and 15 were insignificant between the groups (p>0.05) (Fig. 2).

After the surgical procedure using PRP, the animals were followed for 24 weeks and evaluated for alterations, such as tumour recurrence and metastasis induced by the product in the bed of the surgery. Thus, chest radiography and abdomen ultrasound were conducted to assess metastasis. The results indicated no significant difference between the groups (p > 0.05).

Subsequently to the surgical procedure for tumour resection, the histological processing of the tumour lesion was used to identify malignancy characteristics through the use of the DMBA carcinogenesis inducer. The administration region had tissue destruction with loss of cells, hyperkeratosis, spongiosa, cell pleomorphism, and cell invasion. The histopathological report of the lesions indicated no significant difference (p> 0.05).

After the use of DMBA, the developed lesions were preneoplastic and occurred in 56% of the animals. Only 3% of the lesions were compatible with squamous cell carcinoma. However, the results indicated that carcinogen-inducing DMBA was able to mutate the deoxyribonucleic acid (DNA) of the epithelial cells because it promoted the formation of actinic keratosis (i.e., preneoplastic lesion). In addition, relapse evaluations showed that a neoplasm was present in some animals. This fact reinforces the idea that the carcinogenesis inducer is capable of stimulating DNA mutation and altering cell characteristics.

It is important to emphazise that, at the end of 16 weeks of carcinogenesis induction, the animals presented nodular and ulcerated lesions that were compatible with squamous cell carcinoma. However, the nodulations were small, and it was impossible to conduct a biopsy beforehand for a conclusion, based on the histopathological report. Therefore, as an alternative, the color Doppler elastography technique was used to evaluate the characteristics of the tissue.

Skin integrity analyses indicated a significant difference in tumour recurrence at 20 weeks with nodulation in the group treated with PRP (p = 0.03). However, there was no significant difference between the groups in the other weeks.

**Figure 3:**
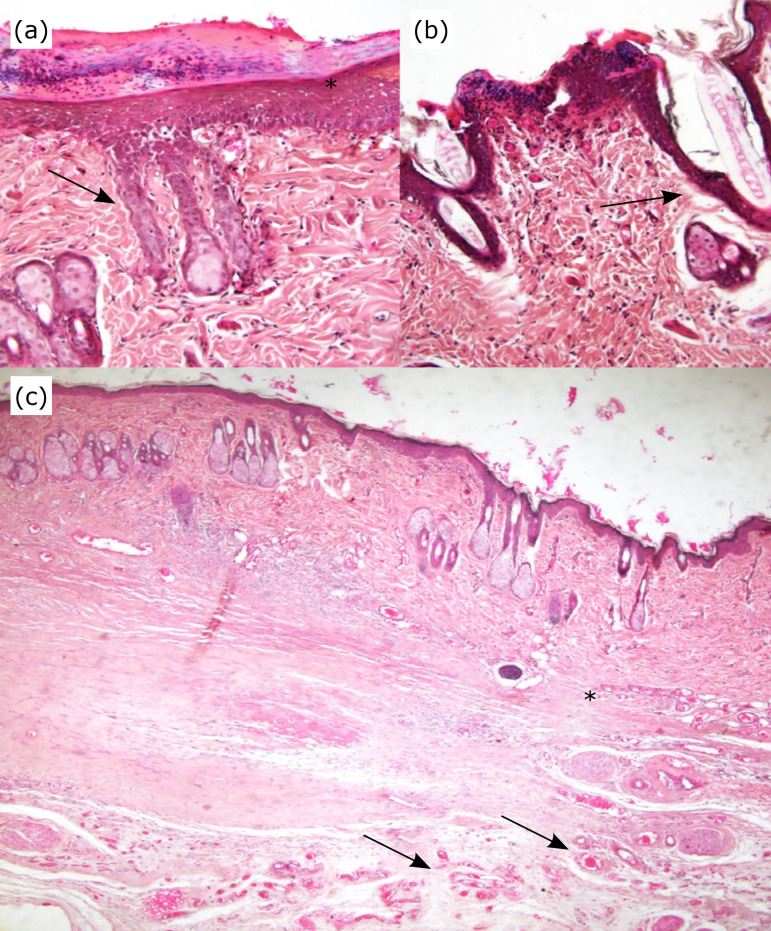
Photomicrography of the epidermis and dermis with malignancy characteristics using the inducer of dimethylbenzantracen (DMBA) carcinogenesis in rats. **(a)** Cell invasion of the spinous layer (*arrow*). An ulceration is in the epidermis (*asterisk*). **(b)** Invasion of the spinous layer (*arrow*). Note the presence of ulceration in the epidermis (*asterisk*). **(c)** Area of tissue destruction, with loss of cell architecture (*asterisk*). Note area with dense collagen and intense vascularization around the lesion (*arrow*). Haematoxylin and eosin; magnification, ×200 [**(a)** and **(b)**] and ×100 **(c)**.

After the 24 weeks, the rats were euthanazised for microscopic evaluations of the site in which the graft was applied. The microscopic evaluation of cicatrization showed no significant differences between the groups regarding the presence of mononucleates, polymorphonucleate, proliferation of fibroblasts, haemorrhage, and necrosis (p> 0.05). The data regarding the scale of epithelial atypia of the surgical scar bed were evaluated by using the criteria proposed by Bánozci *et al*.^29^ and based on the number of criteria for epithelial atypia. The findings were classified as absent, mild, moderate, and intense. The data evaluation indicated that there was no significant difference between the epithelial atypia gradient of the treated and control groups (p> 0.05). The histopathological report of the cicatricial lesions of the surgical procedure did not indicate a significant difference between the groups (p> 0.05). However, it was possible to visualize alterations in the cicatricial epithelium suggestive of pre-neoplastic lesions in the cicatricial bed. In addition, a distal neoplasm was formed in both groups, which was seen macroscopically and confirmed microscopically (Fig. 3).

**Figure 4:**
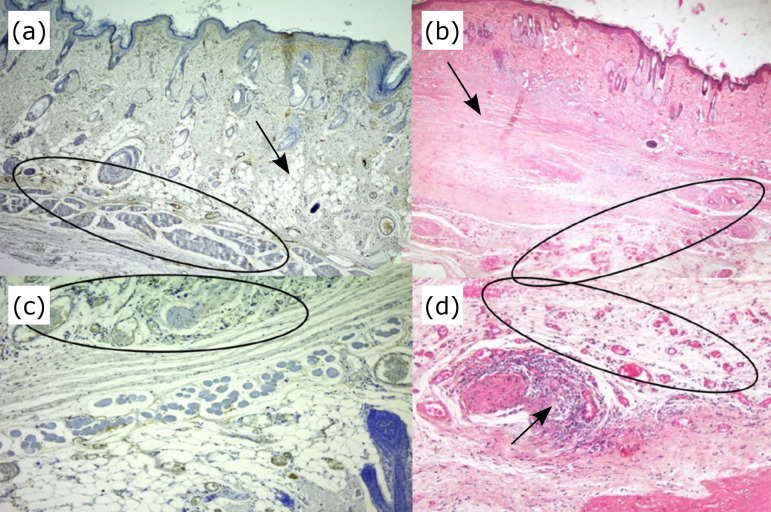
Photomicrographs of vascular proliferation correlated with epithelial atypia. **(a)** Vessels are present, as evidenced by CD31 (*circumscribed area*). An area of the dermis is destroyed (*arrow*). **(b)** Vessels are present, as evidenced by haematoxylin and eosin (*circumscribed area*), notice destruction of the dermis (*arrow*). **(c)** Vessels are present (*circumscribed area*), as evidenced by CD31 (*imunocation*); magnification, ×100. **(d)** Vessels are present (*circumscribed area*) with an inflammatory infiltrate (*arrow*), as evidenced by haematoxylin and eosin; magnification, ×200.

**Figure 5:**
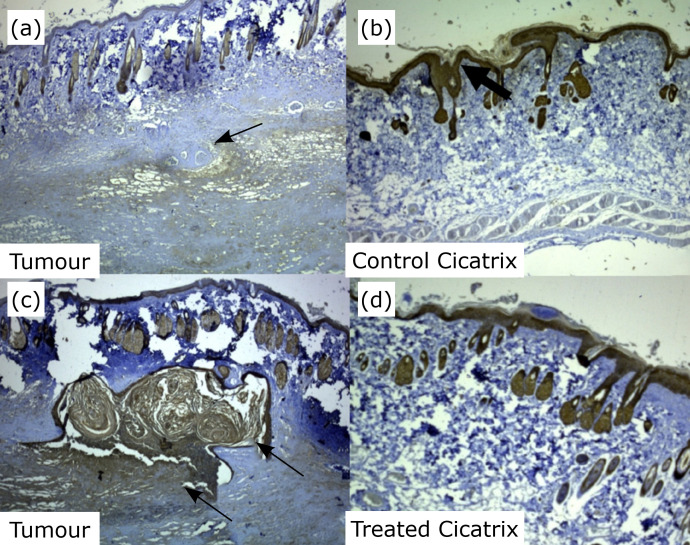
Photomicrography of tumour re-epithelialization and recurrent cicatricial lesion using the Ae1/Ae3 antibody to analyze the percentage of the total area of cell differentiation between the control and platelet-rich plasma (PRP)-treated groups. **(a)** Discrete immunoblot of the epidermis. There is a transition area of the dermis and neoplasm with the presence of discrete immunoblotting (*arrow*). **(b)** Surgical scar in a control group rat shows moderate immunostaining in the epidermis (*arrow*). **(c)** Immunostaining of the dermis and epidermis shows areas with comedo (*arrow*), which is indicative of malignancy. **(d)** Surgical scar of the group treated with PRP with moderate epidermal immunolabeling. Immunolation Ae1/Ae3; magnification, 200.

An analysis of data from CD31 for vascular proliferation and correlation with epithelial atypia indicated that atypia increased with an increase in the vessel variable: the greater the number of the vessels, the greater was the atypia. This finding is an indicator for the progression of a lesion with malignant potential (Fig. 4). As for tumour re-epithelialization and recurrence cicatrization lesion using the Ae1/Ae3 antibody to analyze the percentage of the total cell differentiation area, there was no significant difference between the groups (p> 0.05) (Fig. 5).

## Discussion

There was no difference between healing in the control group and treatment with PRP. However, macroscopic evaluation of the wound indicated it was possible to determine that PRP treatment presented a better aspect of the surgical wound, compared with the control, but without significant difference. Thus, although no significant difference existed, the results of the current study were consistent with those of Pazzini *et al*.^30^, who used PRP gel on cutaneous grafts associating surgical sponges as a compressive dressing and concluded that it favored healing and integration of the graft into the recipient bed without the presence of granulation tissue.

Doppler elastography technique was used to evaluate the characteristics of the tissue. In this technique, shear waves are used to evaluate the elasticity of a tissue, based on increased stiffness; the tissue demonstrated characteristics compatible with neoplasias. However, when the samples were evaluated using histopathology, lesions compatible with squamous cell carcinoma and with actinic keratosis (a characteristic of preneoplastic lesions) were detected. The results of this study indicated that actinic keratosis occurred in most of the animals, but the lesions presented malignant characteristics. These results are consistent with those described by Costa *et al*.^31^, who studied cell proliferation and cyclooxygenase-2 expression as prognostic parameters in actinic keratosis and cutaneous squamous cell carcinoma in dogs. Costa *et al*. found that actinic keratosis and squamous cell carcinoma behave similarly, which justifies the results of this study.

The use of PRP is good in reconstructive surgery, because it stimulates angiogenesis, mitogenesis and chemotaxis, thereby favoring the repair process. However, based on the results in this study, it was possible to determine that its use is contraindicated in patients who undergo tumour resection, as it may cause alterations in the surgical bed, and stimulate local recurrence and metastasis. In this study, rhabdomyosarcoma was diagnosed at a distance from the surgical bed, which indicated that the interaction of PRP in the bed of the surgical wound was capable of promoting neoplasia at a distant site. An interesting finding in this study was that the neoplasm was different from the one initially induced in animals.

The results of this study corroborate what has been reported in the literature^32^, which contraindicate the use of PRP in surgery of cancer patients in human medicine. In addition, patients who received treatment for cancer in a period of five tumour recurrence and metastasis can be reported. However, Dias *et al.* 32 evaluated the association of PRP and bacillus Calmette-Guérin (BCG) in the treatment of noninvasive bladder cancer and concluded that growth factor in PRP had a key role in modulating immune responses, and its association with BCG triggers a better immune response than that of BCG or PRP therapy by itself. It may also be an important therapeutic strategy for noninvasive bladder cancer. A moderate recovery of antigen expression, which immunes system, was similar for both treatments. Such results are extremely important for human and veterinary medicine, because they indicate favorable PRP effects in cancer patients.

About the macroscopic analysis of tumour recurrence, after the surgical procedure in which PRP was administered in the surgical wound bed, the animals were evaluated for 24 weeks to follow the development of possible recurrence and the presence of metastasis^24^. However, the macroscopic analysis of tumour recurrence found no significant difference between the groups in the other weeks.

According to Wright and Dufresne^33^, cellular atypia, which characterizes oral premalignancy, is epithelial dysplasia or carcinoma in situ and it is intense in most cases. In addition, Marcucci^34^ observed in his study numerous cases of cheilitis, described in the literature by solar or actinic incidence. This finding suggests the importance of considering such lesions as carcinogenic, and highlights the scarcity of studies that reveal the percentage of this possible transformation.

These studies show the results found in this research. The lesions diagnosed by the histopathological examination were compatible with squamous cell carcinoma and in most cases with actinic keratosis, and as it was seen and proven there was recurrence in some patients, as well as the presence of new neoplastic lesions at a distance. This fact reinforces the idea that, if the chemically-induced lesions were not malignant, they would not have presented potential for relapses and metastasis, and predispose to the development of other neoplasms after the association with the PRP.

Farrar *et al*.^35^ and Vineet *et al*.^36^, who studied the expression of Ae1/Ae3 in oral squamous cell carcinoma, report that immunoexpression in the respective antibodies was negative in most cases of mild, moderate, and severe dysplasia, except in severe dysplasia in which 50% of the cases had a positively colored basal layer. This fact suggests that cases of dysplasia that did not present Ae1/Ae3 staining in the basal layer may be lesions that are more likely to progress to malignancy. The results of this study are consistent with those in the literature^35^^-^ 37. There was no significant difference regarding immunolabeling of the Ae1/Ae3 antibody, although it can be inferred that, if lesions exist as preneoplastic at the time of surgical intervention, then if disease progression occurred, it would be possible to obtain squamous cell carcinoma compatible malignant lesions. Arnaud *et al*.^38^ found multifocal aspects in their study evaluating the clinical and histopathological aspects of actinic keratosis lesions. It is relevant to emphasize that no clinical features, even if they appear harmless, should be underestimated, because in Arnaud *et al*.’s study^38^ there was no perfect clinical correlation with the histological aspects indicative of dysplasia or squamous cell carcinoma.

## Conclusions

The use of PRP in reconstructive surgeries resulting from resection of neoplasm is contraindicated, because its properties stimulate angiogenesis, mitogenesis, and chemotaxis. The results of this study indicate that pre-neoplastic lesions that appeared in the bed of PRP application have potential for malignancy and development of squamous cell carcinoma. In addition, lesions with less malignancy were sufficient to influence recurrence and predispose to a distant neoplasm. Thus, it can be inferred that, because of its properties, PRP may interact with potential tumour cells that remain in the recipient site of a surgical wound during the resection of a neoplasia, and a local recurrence process can be triggered by the application of this product.
